# Modeling Neurodegenerative Diseases Using *In Vitro* Compartmentalized Microfluidic Devices

**DOI:** 10.3389/fbioe.2022.919646

**Published:** 2022-06-24

**Authors:** Louise Miny, Benoît G. C. Maisonneuve, Isabelle Quadrio, Thibault Honegger

**Affiliations:** ^1^ NETRI, Lyon, France; ^2^ BIORAN Team, Lyon Neuroscience Research Center, CNRS UMR 5292, INSERM U1028, Lyon 1 University, Bron, France; ^3^ Laboratory of Neurobiology and Neurogenetics, Department of Biochemistry and Molecular Biology, Lyon University Hospital, Bron, France

**Keywords:** microfluidic, neurodegenerative disease, *in vitro*, brain-on-chip, Alzheimer’s disease, Parkinson’s disease, amyotrophic lateral sclerosis

## Abstract

The human brain is a complex organ composed of many different types of cells interconnected to create an organized system able to efficiently process information. Dysregulation of this delicately balanced system can lead to the development of neurological disorders, such as neurodegenerative diseases (NDD). To investigate the functionality of human brain physiology and pathophysiology, the scientific community has been generated various research models, from genetically modified animals to two- and three-dimensional cell culture for several decades. These models have, however, certain limitations that impede the precise study of pathophysiological features of neurodegeneration, thus hindering therapeutical research and drug development. Compartmentalized microfluidic devices provide *in vitro* minimalistic environments to accurately reproduce neural circuits allowing the characterization of the human central nervous system. Brain-on-chip (BoC) is allowing our capability to improve neurodegeneration models on the molecular and cellular mechanism aspects behind the progression of these troubles. This review aims to summarize and discuss the latest advancements of microfluidic models for the investigations of common neurodegenerative disorders, such as Alzheimer’s disease, Parkinson’s disease, and amyotrophic lateral sclerosis.

## Introduction

Neurodegenerative diseases (NDD), a type of neurological disorders (ND) involving the central nervous system (CNS) and/or the peripherical nervous system (PNS) degeneration, affect tens of millions of people worldwide, and this number keeps on increasing every year (Mofazzal Jahromi et al., 2019). *In vivo* research using animal models have been an important tool to study both the function of the peripheral nervous system and the pathophysiological mechanisms of NDD. While genetically engineered animals have been the main paradigm to model human diseases for decades, the physiological differences between animals and humans are significant and cannot be ignored (Dauer and Przedborski, 2003; Haring et al., 2017; Osaki et al., 2018a; Ali et al., 2019) an overwhelming quantity of clinical trials have failed because of the lack of translationality between these animal models and humans (Haring et al., 2017; Osaki et al., 2018a; Ali et al., 2019). Besides *in vivo* studies, *in vitro* two-dimensional (2D) and three-dimensional (3D) culture of neuronal cells or tissues have been used to perform neuro-cytotoxicity screening and improve the drug discovery process in the NDD research field (Choi et al., 2017; Osaki et al., 2018a). *In vitro* 3D models especially have enabled the development of more relevant models recapitulating for brain complex functional connectivity patterns (Jorfi et al., 2018). These models, particularly brain organoids, can recreate several intricate features of the CNS and PNS via co-culturing different subtypes of neuronal cells that self-organize into microstructures (Roach et al., 2017; Osaki et al., 2018a; Ali et al., 2019; Nikolakopoulou et al., 2020). Brain organoids can be used to recapitulate the heterogeneity of neural cells, however scaling up the intrinsic 3D organizational complexity of the human brain is limited to only some specific brain regions (Hasan and Berdichevsky, 2016; Park et al., 2018). In these models, the exact positioning of the cells cannot be controlled, leading to low architectural reproducibility and to neural networks constructions that might highly differ from the human brain (Jorfi et al., 2018; Slavin et al., 2021). Thus, there is a lack of physiologically relevant neurological models to study disease pathogenesis and to understand the complexity of neural network architecture and function that microfluidic models could fill.

Microfluidic devices are state-of-the-art research tools that can reconstruct minimalistic human neural circuits for the *in vitro* study of cellular connectivity (Taylor et al., 2005; Kamudzandu et al., 2019). They are also used to create *in vitro* complex neuronal networks allowing co-cultures (Hasan and Berdichevsky, 2016; Park et al., 2018) and control over the directionality of the neuronal connections (Peyrin et al., 2011; Courte et al., 2018), while having the capacity to isolate the soma of the seeded neurons from their neurites (Taylor et al., 2003; Paranjape et al., 2019; Sung, 2021). Such devices are used for the culture and compartmentalization of cells to perform large-scale drug screenings and pharmacological assays in neuroscience, particularly in NDD research (Jadhav et al., 2015; Yi et al., 2015; Neto et al., 2016; Choi et al., 2017; Osaki et al., 2018a; Ali et al., 2019; Tian et al., 2019; Nikolakopoulou et al., 2020).

In this review, we aim to highlight the role of minimalist brain models using this type of microfluidic devices, also called brain-on-chip (BoC), for the study of the cellular and molecular mechanisms of NDD. First, we present an overview of the microfluidic designs used to investigate the human brain. Then, we describe the different microfluidic platforms that modelled some of NDD such as Alzheimer’s disease (AD), Parkinson’s disease (PD), dementia with Lewy bodies (DLB) and amyotrophic lateral sclerosis (ALS).

## Microfluidic Technology: Designs and Methods

Due to the inherent complexity of human neuronal networks, the study of the CNS remains a puzzle to solve. Conventional neuronal cell culture using petri dish leads to the creation of random connections between neuronal cell types which is not representative of human brain physiology (Hasan and Berdichevsky, 2016; Choi et al., 2017; Haring et al., 2017). Utilizing the capability of compartmentalization of microfluidic devices, several researchers used microfluidic technologies to effectively co-culture different neuronal cell types to get closer to the physiology of the human brain (Maschmeyer et al., 2015; Zahavi et al., 2015; Kamudzandu et al., 2019). In comparison to classical cell culture techniques, this technology is cost effective, highly reproductible and allows for flexibility on the structural design of the devices, thus improving cell manipulation and enabling the regulation of cell interconnectivity (Hasan and Berdichevsky, 2016; Osaki et al., 2018a; Kamudzandu et al., 2019; Mofazzal Jahromi et al., 2019). These customized systems also enable users to apply and control mechanical factors such as physical strains and pressure gradients, that are necessary to mimic the physiological conditions of the human brain (Neto et al., 2016; Ahn et al., 2017; Osaki et al., 2018a; Nikolakopoulou et al., 2020). Moreover, since microfluidic facilitates multiplexing, repeatability and reproducibility, while using microvolumes of reagents, high-throughput (HT) drug screening assays can also be performed (Jadhav et al., 2015; Du et al., 2016; Muller et al., 2016a; MacKerron et al., 2017; Fantuzzo et al., 2020; Spijkers et al., 2021).

### Architecture of Micropatterned Systems

In the ‘90s, Robert B. Campenot, pioneered the application of microfluidics for the study of neuronal connectivity. By developing the “Campenot” devices, also called Campenot chambers, he successfully cultured a small number of neurons in one compartment and controlled neurites growth up to a second compartment using nerve growth factors (Campenot, 1977, 1982). This methodology to separate the soma from the neurites was revolutionary for the neuroscience field and many scientists later used Campenot’s base concept to fabricate improved designs taking advantage of the advances in microfabrication. In these improved designs, the compartments, also often called chambers nowadays, are linked by microchannels which are of micrometrical dimensions, effectively allowing the culture of several neural types in a small volume of fluid on the same device (Taylor et al., 2003, 2005; Park et al., 2009b). These microchannels are the mean for the various neural types to connect to one another.

An original compartmentalized microfluidic design to model neuronal connectivity was presented by Taylor et al. (Taylor et al., 2003, 2005) in order to replace the Campenot’s (Campenot, 1977, 1982) for physiological models of neuronal networks *in vitro*. They developed a platform with fluidically isolated compartments to implement physical confinement of neurons into pre-designed locations thanks to microchannels dimensions (3-µm high and 10-µm wide) (Taylor et al., 2003). Neurons are seeded in chamber through reservoirs linked with seeding channels (Table 1; number 1, 2, 3, and 4, in the schematic visualization). Because of the microchannels architecture linking two compartments (Table 1; number 5 and 6, in the schematic visualization), neuronal somas are confined in one chamber while only neurites can pass through the microchannels (Table 1; number 7, in the schematic visualization) into the second chamber. Most compartmentalized microfluidic systems that are used for ND modelling are composed of one or more separated compartments, that are connected via arrays of microchannels (Table 1), enabling a fluidic isolation between compartments while maintaining functional connectivity (Taylor et al., 2003; Dinh et al., 2013). To further enhance and increase the complexity of these connectivity patterns, microfluidic architectures can be designed with multiple linking chambers.

**TABLE 1:** Characteristics and reported applications of different microfluidic devices used in neuroscience research (Kamudzandu et al., 2019). Adapted from Kamudzandu et al. (2019) (copyright Biomedical Physics & Engineering Express) (Peyrin et al., 2011); Extracted and adapted and from Peyrin et al. (2011). (copyright Lab on a Chip) (Lassus et al., 2018); Extracted and adapted from Lassus et al. (2018) (copyright Scientific Reports). Schematic visualization: 1 and 3: inlets; 2 and 4: outlets; 5 and 6: channels; 7: microchannels/microgrooves.

Schematic visualization	1 node	2 nodes	3 nodes	4+ nodes
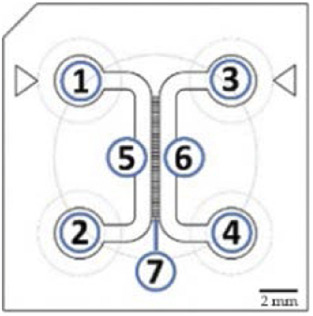	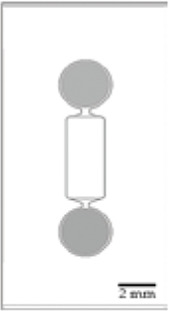	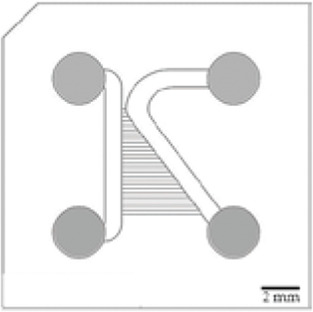	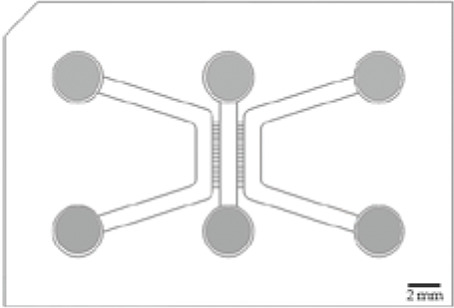	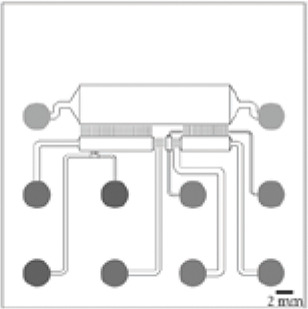
References architectures	Maisonneuve et al. (2021a)	Maisonneuve et al. (2021a)	Maisonneuve et al. (2021a)	Maisonneuve et al. (2021b)
Fabrication methods	3D printing (Amin et al., 2016)	Photolithography (Taylor et al., 2005)	Photolithography (Moutaux et al., 2018; Virlogeux et al., 2018)	3D printing (Amin et al., 2016)
Materials	PDMS	PDMS, COC	PMDS, COC	PDMS
Add-on directionality	N/A	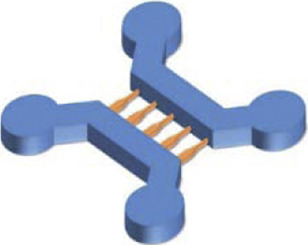 (Peyrin et al., 2011)	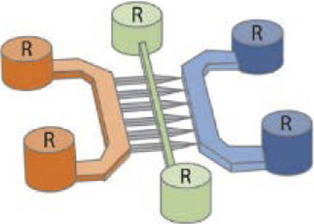 (Lassus et al., 2018)	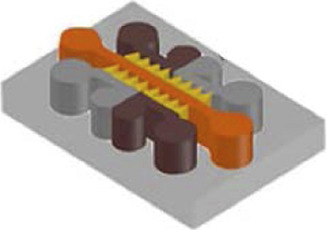 (Kamudzandu et al., 2019)
Applications examples	Functional recording	Co-culture, (ex: Neuron-neuron, NMJ neuro-glial)	Axotomy, synaptic injury	Prion like propagation diseases, functional recordings
Electrophysiology	Fluorescent imaging, MEA, HD-MEA	Fluorescent imaging, MEA	MEA	MEA

Microfluidic systems can also be used to control the mechanical stress applied on cells, by creating a regulated fluid flow *via* hydrostatic pressure which is not present in conventional cell culture (Taylor et al., 2003; Park et al., 2009b). A different pressure between microfluidic compartments leads to flow shifting from one chamber to another. It can be useful to form specific chemical concentration gradients that stimulate many biological processes (Seidi et al., 2011; Shin et al., 2012; Muller et al., 2016b).

The original design can be adapted for other applications. For example, Maisonneuve et al. (2021a) have manufactured a novel triangle-shaped neurofluidic device to study the kinetics of neurite growth (Table 1, 2nodes). Both channels were linked by asymmetric microchannel of various length allowing the accurate monitoring of neurite growth kinetics in a neuronal culture. Maisonneuve et al. (2021b) have also developed a new microfluidic technology, called deposition chamber, that allows to control both the homogeneity and the density of cell seeded while avoiding excessive shear stresses. They fabricated a single compartment design (Table 1, 1 node), where no microchannels are included and can be used to study functional aspects using electrophysiological recording, optogenetics or calcium imaging. Moreover, they used deposition chambers linked with classical microchannels, to create a model of a CNS network affected in NDD: the direct way of basal ganglia (BG) loop, which is known to be involved in PD and HD. This model is composed of five compartments interconnected with microchannels, representing each anatomical region involved in BG loop and respecting the cell number ratios between these brain regions (Table 1, 4+ nodes) (Maisonneuve et al., 2021b).

Scientists can also improve compartmentalized designs by introducing axonal guidance systems that applied oriented directionality to the developing neurites (Peyrin et al., 2011; Schneider et al., 2013; Courte et al., 2018; Holloway et al., 2019; Nikolakopoulou et al., 2020; Vakilna et al., 2021). One of the microchannels architecture is the axonal diodes which are asymmetrically shaped with large opening areas on one end (15 µm wide) and small opening areas on the other end (1–3 µm wide) (Peyrin et al., 2011). They allow neurites growth from one compartment to the other because they can pass through the large opening and not the small one, creating a polarity system. Holloway et al. (2019) compared different structures enabling directionality outgrowth. In addition to the “axonal diodes” architecture, they have investigated microchannels architectures using loop back structures developed by Renault et al. (2016). These structure designs are based on prohibitive and permissive edge guidance paths with repetitive shaped motifs, called arch (Courte et al., 2018), pretzel, heart and arrow head (Holloway et al., 2019).

### Microfabrication Techniques and Materials

To develop 2D and 3D microfluidic systems that can recreate the *in vivo* human microenvironment, the materials and technical approaches used for their fabrication must be carefully considered. Microfluidic devices used for neuroscience research are usually fabricated using a combination of SU-8 photolithography and soft lithography techniques (Campenot, 1977; Park et al., 2006). Recently, 3D printing techniques to produce microfluidic devices have gained importance, since their ability for a faster and lower-cost fabrication makes them an attractive alternative to conventional microfabrication protocols (Kundu et al., 2020). These techniques allowed the fabrication of microfluidic device’s chambers and microchannels (Ren et al., 2013). Bioprinting has recently been applied to microfluidics as well (Haring et al., 2017), enabling the deposition of biological materials and cells into a specific 3D organization for the assembly of tissue scaffolds. This innovative technology has already been used to develop several microfluidic organ models (Yu et al., 2019) but, to our knowledge, there is still no developed BoC model using bioprinting.

Currently, the most common material to manufacture microfluidic devices are usually fabricated is an elastic silicon-based polymer called polydimethylsiloxane (PDMS), offering many advantages such as being nontoxic, biocompatible, transparent and permeable to oxygen which is important for engineering BoC models (Yang, 2010; Neto et al., 2016; Ali et al., 2019; Jiang et al., 2019; Nikolakopoulou et al., 2020). Gas permeability allows the long-term culture of cells for the study of neuronal physiopathology (Taylor et al., 2003, 2005; Hasan and Berdichevsky, 2016; Nikolakopoulou et al., 2020) and transparency facilitates the examination of several cellular features by enabling the use of different optical and fluorescent microscopy techniques (Park et al., 2015; Haring et al., 2017; Nikolakopoulou et al., 2020). Although all these characteristics make the PDMS-based systems one of the most used materials for microfluidic devices fabrication, they have some limitations, such as their tendency to absorb some molecules via non-specific binding, thus affecting locally the concentration of specific cellular components or drugs (Rodrigues et al., 2017; Mofazzal Jahromi et al., 2019; Nikolakopoulou et al., 2020). Other microfabrication materials have been investigated to be used as potential alternative to PDMS, including transparent thermoplastics like poly(methyl methacrylate) (PMMA), polycarbonate (PC), polyetherimide (PE) and cyclic olefin copolymer (COC). These biocompatible materials have reduced absorption of molecules although they show a lower permeability to oxygen (Park et al., 2009a; Kortmann et al., 2009; Gencturk et al., 2017; Haring et al., 2017; Rodrigues et al., 2017; Yu et al., 2019).

Several companies have commercialized a numerous of microfluidic devices for neurosciences research and brain models (Table 2). Xona microfluidics, co-founded by Anne Taylor who has developed microchannels technology with fluidic isolation, proposes microfluidic devices with COC allowing microfluidic industrialization. MicroBrainTech, Ananda and NETRI have developed their microfluidic devices using PDMS. NETRI for example has successfully developed complex BoC with neurofluidic devices ranging from one to five compartments, with and without microchannels. AIM Biotech has used some thermoplastics for manufacturing devices that can be used for cell migration studies with addition of gel scaffolds. They have developed a 3D blood-brain barrier (BBB) model created by a gel interface with a neurovascular unit. Mimetas has developed hydrogel compatible microfluidic devices for extracellular matrix models as well. Synvivo has also developed a BBB model, using a polycarbonate membrane in their devices with two or three compartments. Emulate, as well, has manufactured two and three compartments’ devices including a membrane for neuronal and endothelial cells co-culture. AxoSim has developed a single and two compartments’ devices used as a nerve on a chip model, allowing the seeding of an explant or an organoid (mini-brain organoid).

**TABLE 2:** Microfluidic devices providers with the different materials used for manufacturing.

Companies	Devices		Applications
	Architecture	Materials	
AIM Biotech	3 compartments	TP	Gel compatible devices for co-culture models (cell invasion and migration, vascular functions…). BBB model
Ananda	2 compartments with microchannels	PDMS	Co-culture, high-throughput assays
AxoSim	1 and 2 compartments	PEG	Nerve on a chip model Mini-brain organoid
Emulate	2 and 3 compartments with membrane	PDMS, membrane	Co-culture neurons-endothelial cells
MicroBrainTech	2 and 3 compartments with microchannels and axonal diodes	PDMS	Axonal directionality for unidirectional neuronal network models
Mimetas	2 and 3 compartments with gel interface	PS and glass	Hydrogel compatible for ECM models
NETRI	1 to 5 compartments with/without microchannels or membrane	PDMS, PS, membrane	Co-culture, NDD models, Network architectures, Interface models, high-throughput assays
SynVivo	3 compartments with membrane	PDMS, membrane	BBB model
Xona	2 and 3 compartments with microchannels	COC and silicone	Axonal isolation

COC, Cyclic olefin copolymer; PDMS, Polydimethylsiloxane; PED, polyethylene glycol; PS, Polystyren; TP, Thermoplastic.

Microfluidic devices are mostly fabricated onto glass or polymer and are bonded in an either reversible or irreversible way. Reversible bonding usually consists of placing the device on a glass coverslip previously coated for cell culture, while irreversible bonding involves the activation of both contacting surfaces though air or oxygen plasma (Zhang et al., 2010; Muller et al., 2016a; Farnese et al., 2021). Most of the devices are fabricated by irreversible bonding in order to accommodate higher pressures and stresses (Neto et al., 2016).

### Cell Culture Interactions in Microfluidic Devices

Whether by bonding microfluidic device to a substrate pre-coated with poly-L-lysine (PLL) or poly-D-lysine (PDL) or by coating the substrate directly in the bonded device, it is possible to use coatings that improve cellular adherence and survival on substrate in 2D culture (Taylor et al., 2005; Park et al., 2006; Neto et al., 2016; Nikolakopoulou et al., 2020). To obtain a closer resemblance to *in vivo* brain microenvironments, biomaterials mimicking the extracellular matrix (ECM) can also be integrated into 2D and 3D microfluidic devices as scaffolds. Neural cells and spheroids can be introduced into a suspension of hydrogel or ECM materials, such as collagen, and incorporated into the device. This system allows 3D culture for the study of angiogenesis or cell migration (Muller et al., 2016a; Lancaster et al., 2017), for example. A more recent study of Fannon et al. (2021), has used hydrogels for *in vitro* 3D cell culture. They have developed a 3D culture platform allowing neural tube development and model.

The human nervous system contains approximately 100 billion neurons, which cluster into specialized populations and extend multiple neurites among the different brain areas to create neuronal circuits that enable the processing and the storage of information (Park et al., 2013b; Jadhav et al., 2015; Choi et al., 2017; Mofazzal Jahromi et al., 2019). To form such circuitry and make connections, neurons in each population are polarized, meaning that their neurites differentiate into either an axon or dendrites to either send or receive electrical information, respectively. Several types of cells can be seeded in microfluidic devices. All classical *in vitro* cell culture approaches for studies of NDD can be reproduced in microfluidic devices. Animal can be a common source of brain cells for *in vitro* cultures. These cells are usually extracted from different anatomical regions of the CNS and PNS of embryos, pups or adult rodents before being seeded into compartments of microfluidic devices (Kunze et al., 2011; Seibenhener and Wooten, 2012; Southam et al., 2013; Robertson et al., 2014; Ruiz et al., 2014; Hallinan et al., 2019). Despite the advantages of using animals, this approach has some limitations. As there are structural differences between animal and human brains, not all types of neural cells can be obtained using this method: not all brain regions are reachable in animals. Moreover, biological differences appear to be problematic as well, since several studies have identified that drug testing results on cells acquired from animal models are poorly relevant when compared with the results of human clinical trial (Dauer and Przedborski, 2003; Choi et al., 2017; Kankala et al., 2018). To obtain a more reliable research system, closer to the human brain, scientists have recently started to integrate human-derived cells into microfluidic devices for the study of CNS or PNS physiology and of NDD pathologies (Ohtani-Kaneko et al., 2017; J; Siney et al., 2018; Sances et al., 2018). Human induced-pluripotent stem cells (hiPSCs) can be differentiated into cell types of interest while maintaining the endogenous genomic background (Choi et al., 2017; J; Siney et al., 2018; Kankala et al., 2018).

Neurons require to interact with glial cells to maintain intercellular connectivity (Harada et al., 2016; Takata-Tsuji et al., 2021). Glial cells are the most numerous neural cell type in the CNS, regulating brain homeostasis through both the structural and functional support of neurons and their role as immune cells (Yi et al., 2015; Neto et al., 2016; Osaki et al., 2018a). Several studies used 2D compartmentalized microfluidic devices to accurately model neuron-glia communication, including synapse formation, function, axonal myelination and signalling (Park et al., 2009b; Hosmane et al., 2010; Majumdar et al., 2011; Shi et al., 2013; Robertson et al., 2014). In 2018, Park et al. went one step further into the research of neuron-glia interaction by developing a human AD model by using a 3D microfluidic platform for the co-culture of neurons, astrocytes, and microglia (Park et al., 2018). Particularly, this design mimicked the recruitment of microglia and astrocytes hyperactivation under AD conditions, allowing for the reconstruction of a complex pathophysiological environment that could be used as an inflammatory or a pharmacological model (Izzo et al., 2014; Vazin et al., 2014).

Besides neuron-neuron and neuron-glia interactions, the study of the neuromuscular junction (NMJ) should also be considered, specifically in the context of NDD affecting motor neurons. The NMJ is composed by the synaptic interactions between neurons from the CNS and myocytes, which role is to induce muscle contractions. The analysis of such interactions helps the understanding of the pathogenesis such as ALS (Zahavi et al., 2015; Jongh et al., 2021). The current NMJ models are based on microfluidic devices containing two or three compartments, allowing the co-culture of motor neurons and myocytes (Park et al., 2013a; Zahavi et al., 2015; Santhanam et al., 2018). These models were able to recapitulate for the neuron-myocyte characteristic interactions and axonal isolation, thus enabling the establishment of drug-screening platforms for NMJ dysfunctions (Osaki et al., 2020).

### Electrophysiological and Functional Activity Recordings in Microfluidic Devices

The functional communication among neurons within a circuit through axonal transmission is a biological process difficult to monitor with classical non-compartmentalized neuronal cultures (Park et al., 2013b; Choi et al., 2017; Osaki et al., 2018a). Neuronal network analysis is possible within microfluidic systems (Maisonneuve et al., 2021a). They thus allow the monitoring of both structural and functional aspects of neuronal culture, making possible to study the impact of a compound applied in one compartment, whose effects are observed in the others (Johnstone et al., 2010; Delamarche et al., 2013).

Neurons’ functional activity can be recorded by different setups compatible with microfluidic devices (Holloway et al., 2021). Microelectrode array (MEA) is a technique that can be placed perfectly under microfluidic devices’ chambers and using micro electrodes that are in direct contact with neurons in culture, have been usually used by the microfluidic community (Bruno et al., 2020; Holloway et al., 2021). This technology allows extracellular recordings, unlike patch clamps which allow only single cell recording and are rarely used in combination with microfluidics (Kamudzandu et al., 2019; Holloway et al., 2021). Fluorescent-based techniques for live-cell imaging and monitoring of neuronal functional activity (Holloway et al., 2021). Calcium imaging is fully compatible with PDMS-based microfluidic devices because of their transparency. Fluorescent calcium probes, such as Fluo-4, Fura-2, and BAPTA-1, as well as calcium voltage indicators, such as GCaMP6f, allow the visualization of intracellular calcium changes as an indication of neuronal functional activity (Robertson et al., 2014; Lassus et al., 2018). However, since the use of calcium imaging is limited to the microscopic scale, only a small group of neurons can be monitored simultaneously, thus preventing the recording of the entire neuronal network.

Nevertheless, the combination of both MEAs and calcium imaging with microfluidic technology opened interesting opportunities to record a complete picture of the *in vitro* functional connectivity of neural circuits at high spatial and temporal resolutions, as exemplified by Moutaux et al. (2018). Using a three-chamber microfluidic device, they seeded two separated neuronal populations in the two distal compartments, defining the central one as the axon-dendrite communication compartment. They then precisely characterized synaptic signal transmission by simultaneously monitoring intracellular calcium dynamics and the pre- and post-synaptic electrophysiological activity (Moutaux et al., 2018).

## Neurodegenerative Disorders-On-A-Chip: Microfluidic Devices for the Study of neurodegenerative Diseases

For decades, the main challenge of neuroscience research in drug development has been the lack of translationality from preclinical studies to human, the preclinical trials usually performed being classical cell cultures and *in vivo* animal modelling. Unfortunately, preclinical results have showed little significance when compared with outcomes in humans; primary because of physiological differences between animals and humans (Syama and Mohanan, 2021). In the context of neurodegeneration, the development of BoC enabled important advances in drug discovery as relevant *in vitro* brain models (Neto et al., 2016; Choi et al., 2017; Haring et al., 2017). Microfluidic devices have demonstrated their usefulness for toxicity assays, pharmacological tests, and drug screening on various cell types, including neurons (MacKerron et al., 2017). For example, several studies have successfully investigated the pathological process within injury using compound application in microfluidic devices developed for neurological disorders (Seidi et al., 2011; Deleglise et al., 2013, 2014; Southam et al., 2013; Ruiz et al., 2014; Tran et al., 2014; Gribaudo et al., 2019; Courte et al., 2021). Thanks to their ability to isolate neurites from somas, microfluidic devices have also been used to study axonal regeneration after mechanical or chemical axotomy (Taylor et al., 2005; Deleglise et al., 2013; Tong et al., 2015; Lyu et al., 2017; Shrirao et al., 2018)**.**

NDD critically affect neuronal function, leading to the progressive loss of neurons in specific regions of the brain and the disruption of the neural network integrity (Yi et al., 2015; Busche and Konnerth, 2016; Choi et al., 2017; Osaki et al., 2018a; Fortanier et al., 2019; Mofazzal Jahromi et al., 2019). An effective curative treatment for such disorders is still a challenge, since the molecular mechanisms causing cellular degeneration are still hypothetical (Cutsuridis and Perantonis, 2006; Calsolaro and Edison, 2016; J; Siney et al., 2018; Natarajan et al., 2019). To overcome this lack of knowledge, it is crucial to closely study the alterations occurring in the neuronal microenvironment. Microfluidic devices, and more especially BoC systems, have improved the collective knowledge in the field of NDD thanks to the minimalistic *in vitro* platforms that enabled the investigation of cellular mechanisms in diseases such as AD, PD and ALS.

In the next sections, we discuss the currently available microfluidic devices fabricated and used for the study of NDD and their main outcomes, also summarized in Supplementary Table S1.

### Alzheimer’s Disease

AD is the most common NDD worldwide, with a prevalence of approximately 10%–15% of people above 80 years old (Morley et al., 2018; Fish et al., 2019; Lewis and Spillane, 2019). It is considered a sporadic disease whose causes have still yet to be identified, although 1%–5% of AD cases show some degree of heredity (Morley et al., 2018; Guest, 2019; Lewis and Spillane, 2019). AD is mainly characterized by two pathological hallmarks, which are the extracellular accumulation of β-amyloid (Aβ) plaques and the intracellular aggregations of hyperphosphorylated Tau protein known as neurofibrillary tangles (NFTs), and are often referred to “amyloid cascade hypothesis” in the literature (Karran and De Strooper, 2016). They cause neuronal damage, leading to loss of synaptic connections and cell death in the human brain (Braak et al., 2006; Alonso et al., 2008; Minter et al., 2016; Guest, 2019).

**FIGURE 1:**
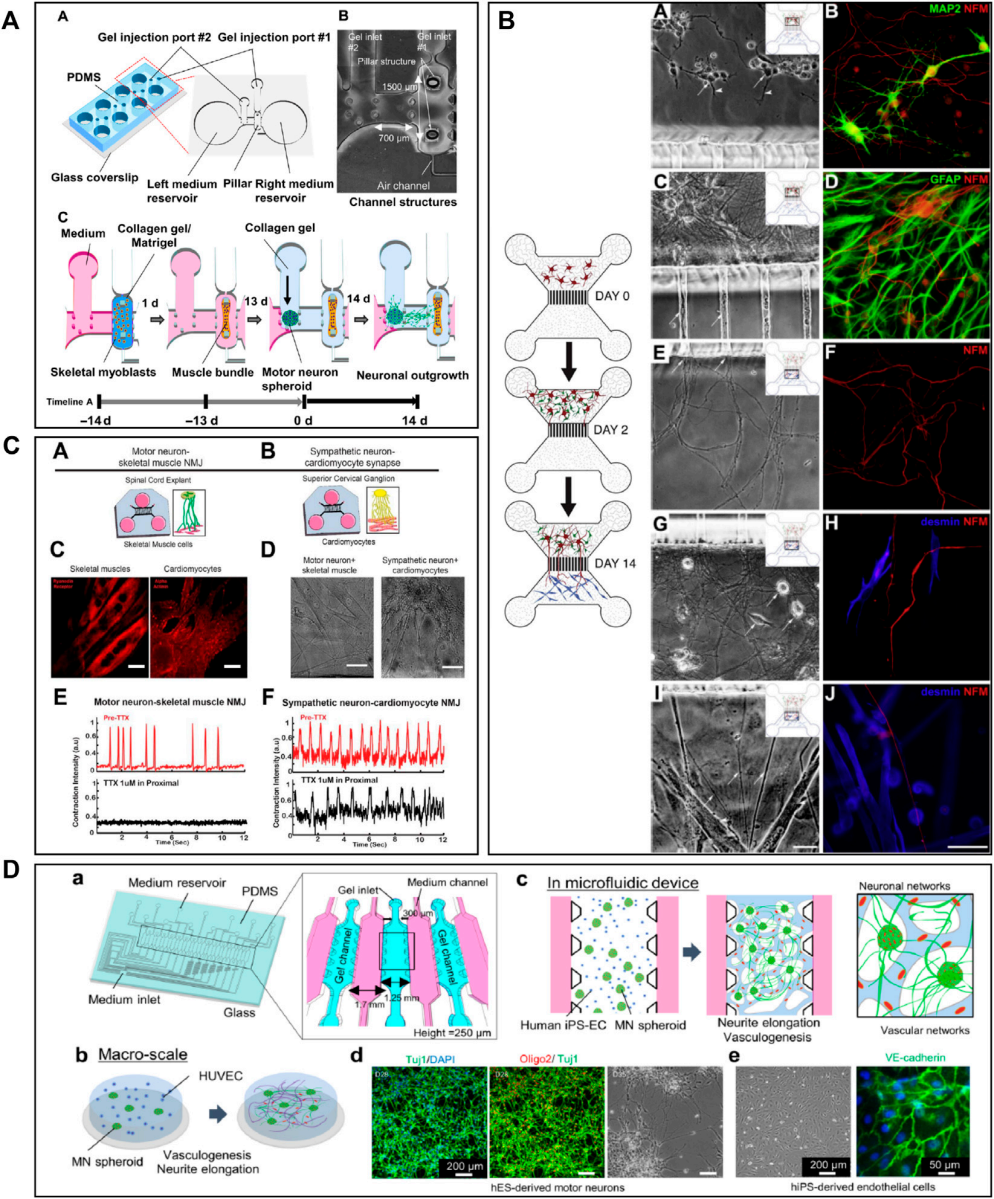
Microfluidic devices used in Alzheimer’s disease modelling. **(A)** Generation of a co-culture of neurons with disease induction using 3 nodes compartmentalized chip with reservoirs to study molecular schematization of inhibitor effect of okadaic acid (OA) on dephosphorylation of Tau-microtubule-binding. Extracted from Kunze et al. (2011) (copyright Willey Periodicals). **(B)** Design of multi nodes compartmentalized microfluidic device and immunofluorescence pictures (βIII-tubulin (red) for axonal and MAP2 (green) for somatodendritic staining, Hoechst (blue) for cell bodies, Synaptophysin 1 (yellow) for presynaptic, Homer 1 (cyan) for postsynaptic sainting, MAP2 (magenta) for dendrites) of presynaptic (pre), postsynaptic (post) and synaptic chamber as well as co-culture chamber with non-neural cell types. Extracted from Kilinc et al. (2019) (copyright Oxford University Press—Brain). **(C)** 3D microfluidic platform with AD neurons, astrocytes h-NPCs-derived and human adult microglia in culture for organotypic human AD modelling. The platform quantifies microglial engagement using immunofluorescent pictures [AD neurons/astrocytes (green), microglial cells (red) and nuclear staining (white)]. Scale bars of picture d and e represent 250 µm. Scale bars for picture f and g represent 150 µm. Extracted from Park et al. (2018), (copyright Nature Neuroscience).

Microfluidic AD models have been fabricated with the goal to accurately analyse the molecular mechanisms leading to the pathology (Figure 1). Many studies attempted to recapitulate the formation of Aβ plaques and NFTs and to evaluate their propagation by examining axonal transport in microfluidics (Stoothoff et al., 2009; Lee and Park, 2010; Kunze et al., 2011; Poon et al., 2011; Deleglise et al., 2014; Dujardin et al., 2014; Song et al., 2014; Calafate et al., 2015; Takeda et al., 2015; Brahic et al., 2016; Katsikoudi et al., 2020). Deleglise et al. (2014) used a microfluidic device composed of two compartments to recreate an *in vitro* cortico-striatal neuronal network model allowing to evaluate the toxic effect of Aβ. Using diodes-shaped microchannel to force unidirectional growth of neurites, they injected Aβ peptide into the neurites compartment and observed a dying-back process in the soma compartment. Song et al. (2014) studied neuron-to-neuron Aβ transmission using microfluidic devices composed of two and three compartments. With these microfluidic architectures, they showed that Aβ was internalized by distal neurites and retrogradely transported to neuronal cell bodies. Other research groups, like Takeda et al. (2015), Calafate et al. (2015) and Nobuhara et al. (2017), used microfluidic devices with three compartments to seed two distinct neuronal populations either into the distant compartments (Calafate et al., 2015; Takeda et al., 2015), using the central one as a synaptic compartment, or in one distant and the central compartment (Nobuhara et al., 2017). They studied Tau propagation between neurons, demonstrating that the spreading of Tau was facilitated with the presence of synaptic contacts (Takeda et al., 2015), and examined the proprieties of different Tau isoforms along neurites (Calafate et al., 2015). Nobuhara et al. (2017) also showed that a specific Tau antibody could influence peptide aggregation and neuronal propagation thanks to this microfluidic architecture. Kunze et al. (2011) used a device with three compartments to generate neurite synapses in the central compartment from two distinct neuronal population. They used okadaic acid (OA), to promote Tau hyperphosphorylation, in the one distal compartment and analysed the connection between the diseased and the healthy neuronal populations (Figure 1A). Other studies used microfluidic devices composed of two or three isolated compartments to investigate tau peptide transport and propagation along neurons using animal cells (Wu et al., 2013, 2016) or hiPSC-derived cortical neurons (Usenovic et al., 2015; Wu et al., 2016).

The design of compartmentalized microfluidic systems for the isolation of soma, neurites and synapses is not only an advantage for the assessment of peptide propagation between neurons, but also for the study of their neurotoxic effect. In Ruiz et al. (2014) fabricated a gravity-induced flow microfluidic device to analyse Aβ toxicity in different neuronal populations seeded in four distinct compartments linked by four streams. They showed that FTY720, a drug used for the treatment of ALS, had a neuroprotective effect. In Kilinc et al. (2019) presented an AD synaptotoxicity model by using a three-compartmentalized device that permitted the isolation of presynaptic and postsynaptic neuronal regions. With this model, they exposed cell cultures to Aβ toxic peptides at low concentrations and analyzed their effects in the synaptic compartment (Figure 1B). Several researchers took advantage of microfluidic designs with multiple compartments for the assessment of the Aβ toxic effect on several neuronal regions. For example, Li et al. (2017) demonstrated that Aβ-induced neurotoxicity depended on its primary localization. By utilizing a three compartments device attached to a constant Aβ perfusion system, they detected that Aβ application on cell bodies and axonal terminals induced neuronal death, but not when applied on dendritic neurites.

Besides the importance of the neurotoxic effect of peptides, neuroinflammation also seems to play a fundamental role in the pathogenesis of AD (Lewis and Spillane, 2019). The immune response in the CNS, and the subsequent neuroinflammation, are mediated by the activation and recruitment of astrocytes and microglia, which contribute to neuronal loss through the secretion of pro-inflammatory cytokines (Calsolaro and Edison, 2016; Minter et al., 2016; Choi et al., 2017). Therefore, in addition to the recreation of the inclusion of mechanisms of inflammation into microfluidic devices has also been a key goal for the construction of a comprehensive AD model. In Park et al. (2018) designed a 3D circular microfluidic system to model AD by co-culturing neurons, astrocytes, and microglia. Thanks to their platform, AD molecular and cellular hallmarks could be studied simultaneously in one single device, including Aβ aggregation, Tau hyperphosphorylation, and neuroinflammation induced by the recruitment of microglia into the neuronal compartment (Park et al., 2018) (Figure 1C). Cho et al. (2013) aimed to study microglial accumulation in response to maintained gradients of Aβ. To do so, they developed a microfluidic chemotaxis platform and found that soluble Aβ could send recruiting signals to microglia, inducing their migration towards the central microfluidic compartment (Cho et al., 2013).

### Parkinson’s Disease and Lewy Body Dementia

PD is the second most common neurodegenerative disease worldwide, presenting a prevalence of approximately 4% of people above 80 years old (Dauer and Przedborski, 2003; Dexter and Jenner, 2013; Stoker and Greenland, 2018; Reich and Savitt, 2019). From an etiological point of view, PD can be described as a multifactorial disorder which main causes range from genetic to environmental factors (Reich and Savitt, 2019). The main neuropathological hallmark associated with PD is the loss of midbrain dopaminergic neurons from the substantia *nigra* (Stoker and Greenland, 2018; Reich and Savitt, 2019). A major consequence of this neuronal loss is the disruption of the dopaminergic signaling from the basal ganglia to the rest of the brain, which leads to the impaired control of the human body motor movements (Dauer and Przedborski, 2003; Stoker and Greenland, 2018; Reich and Savitt, 2019). It is believed that this neuronal loss is associated to the development of intracellular protein-rich inclusions known as Lewy bodies, whose main component is aggregated α-synuclein (Braak et al., 2003, 2004). One of the most studied molecular mechanism of PD [also involved in other synucleinopathies like Lewy body dementia (LBD)] is the propagation of misfolded α-synuclein between neurons, as it is prone to aggregate and promote the initiation of a neurotoxic process (Braak et al., 2003; Delenclos et al., 2017; Sanford, 2018).

**FIGURE 2:**
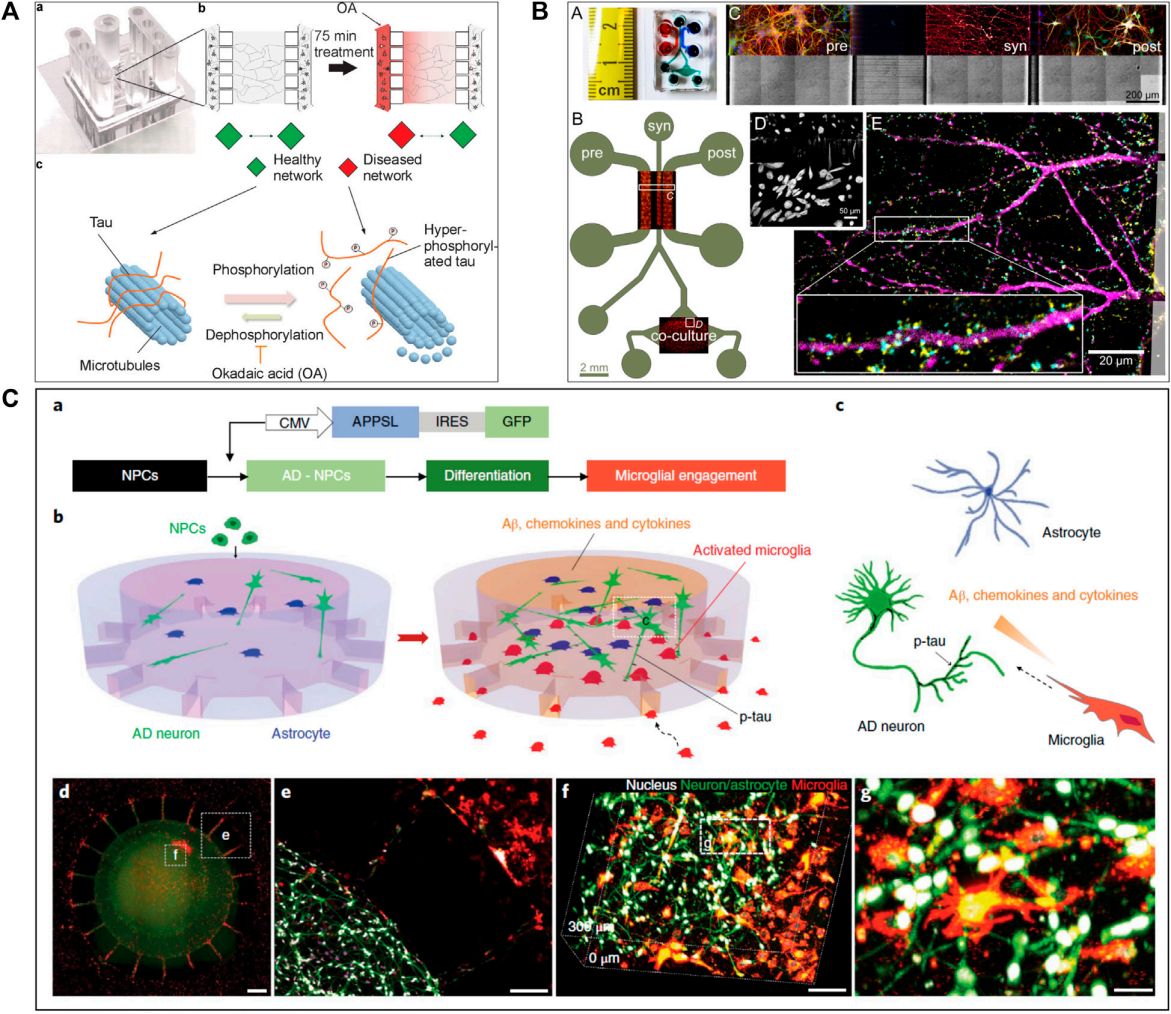
Microfluidic devices used in Parkinson’s disease modelling. **(A)** Representation of α-synuclein assemblies spreading from proximal chamber to distal chamber of microfluidic device with 3 compartments, thanks to immunofluorescent staining. Scale bars represent 25 µm. Extracted from Gribaudo et al. (2019) (copyright Stem Cell Reports). **(B)** Schematic representation of the experiment with **(C)** and without **(A)** neurons, stained by βIII-tubulin in red adding α-synuclein fibrils stained by Syn-488in green, in first compartment of the microfluidic device. Extracted from Freundt et al. (2012) (Copyright Annals of Neurology). **(C)** Representation of *in vitro* microfluidic device of basal ganglia circuit with five compartments representing anatomical region and connectivity. Neuronal co-culture was characterized by immunofluorescent pictures of cortical (V-glut in green), striatum (GABA in red), globus pallidus (PY in green) and substantia nigra (TH in red) neurons. Extracted from Kamudzandu et al. (2019) (Copyright Biomedical Physics & Engineering Express).

Several studies (Seidi et al., 2011; Freundt et al., 2012; Tran et al., 2014; Cavaliere et al., 2017; Gribaudo et al., 2019) attempted to create a precise *in vitro* PD and LBD microfluidic model by recreating toxic α-synuclein spreading (Figure 2). For example, Tran et al. (2014) used a three compartments microfluidic device to show that α-synuclein monoclonal antibodies could diminish Lewy body formation *in vitro*, and thus prevent neuronal death. They could demonstrate a reduction of neuron-to-neuron α-synuclein propagation, leading to a decrease in peptide fibril formation and uptake (Tran et al., 2014). Freundt et al. (2012) presented a neuronal culture model to study α-synuclein spreading mechanisms. Their microfluidic device was composed of two compartments linked with microchannels, where fibrils of α-synuclein were injected into the axonal compartment. They showed that α-synuclein was internalized by neurons, transported along axons, and then released from neurons into the extracellular media (Figure 2B). Volpicelli-Daley et al. (2011) investigated the toxic effect of endogenous α-synuclein aggregates that propagated along neuronal axons up to synapses. They showed that the accumulation of aggregated α-synuclein leads to severely injured synaptic connections and impaired neuronal excitability.

To increase the pathophysiological relevance of the microfluidic PD models, Gribaudo et al. focused on the integration of hiPSC-derived neurons into a microfluidic device including three compartments interconnected with asymmetrical diode-shaped microchannels (Gribaudo et al., 2019) (Figure 2A). Using this design, they studied the prion-like propagation of different α-synuclein forms, such as monomeric and fibrillar and showed that its accumulation affected synaptic integrity and mitochondrial morphology. Considering the importance of mitochondrial involvement in the pathogenesis of PD, novel technologies were developed to improve mitochondrial visualization along neurons. For example, Lu et al. (2012) developed a two compartments microfluidic device linked by microchannels for the specific visualization of mitochondrial transport along single axons by live-cell imaging using confocal microscopy, allowing axonal staining through microchannels.

In PD and LBD, neuroinflammation with glial cells and astrocytes involvement is a significant consequence of the neuropeptide propagation leading to neurodegeneration (Wang et al., 2015; Sarkar et al., 2020). To study interactions between neurons and astrocytes, Cavaliere et al. (2017) used a two compartments microfluidic device for the co-culture of different neural cell combinations, to assess the transport and propagation of α-synuclein aggregates. They could show that α-synuclein spreading from astrocytes to neurons can lead to neuronal dysfunction and degeneration.

Two recent studies (Kamudzandu et al., 2019; Maisonneuve et al., 2021b) have used microfluidic technology to develop a BG loop model deeply involved in Parkinson’s disease. Kamudzandu et al. (2019) developed a complex *in vitro* brain circuitry as “organ-on-chip” devices modelling BG. Using five compartments linked with unidirectional diode-shaped microchannels in a microfluidic device, they co-cultured the specific neuronal subtypes constituting the BG using rodent primary neurons. They have shown a direct connectivity and network communication across a five-population neuronal circuit (Figure 2C). In another study, Maisonneuve et al. (2021a) used neurofluidic technology to create a model of the direct way of BG loop of the brain, involved in Parkinson disease and Huntington disease, composed of five interconnected compartments. Each nodes represented an anatomical region involved in BG loop and were aligned on the device with respect to the cell number ratios between the various brain regions, linked with microchannel (Table 1) (Maisonneuve et al., 2021b).

### Amyotrophic Lateral Sclerosis

ALS is a NDD that specifically involves the loss of spinal motor neurons, which control the voluntary contraction of muscles. For the most case, ALS is a sporadic disease, with familial type representing 5%–10% of the cases (Arjmand et al., 2022). There are several theories explaining its pathology and progression. The first one is the “dying-forward” hypothesis and is based on a disorder of both motoneurons and cortical neurons. The second one is the “dying-back” hypothesis, which involves the muscle cells or the NMJ. The third hypothesis proposes that the upper and lower motor neuron degeneration occur independently from each other (Valko and Ciesla, 2019).

**FIGURE 3:**
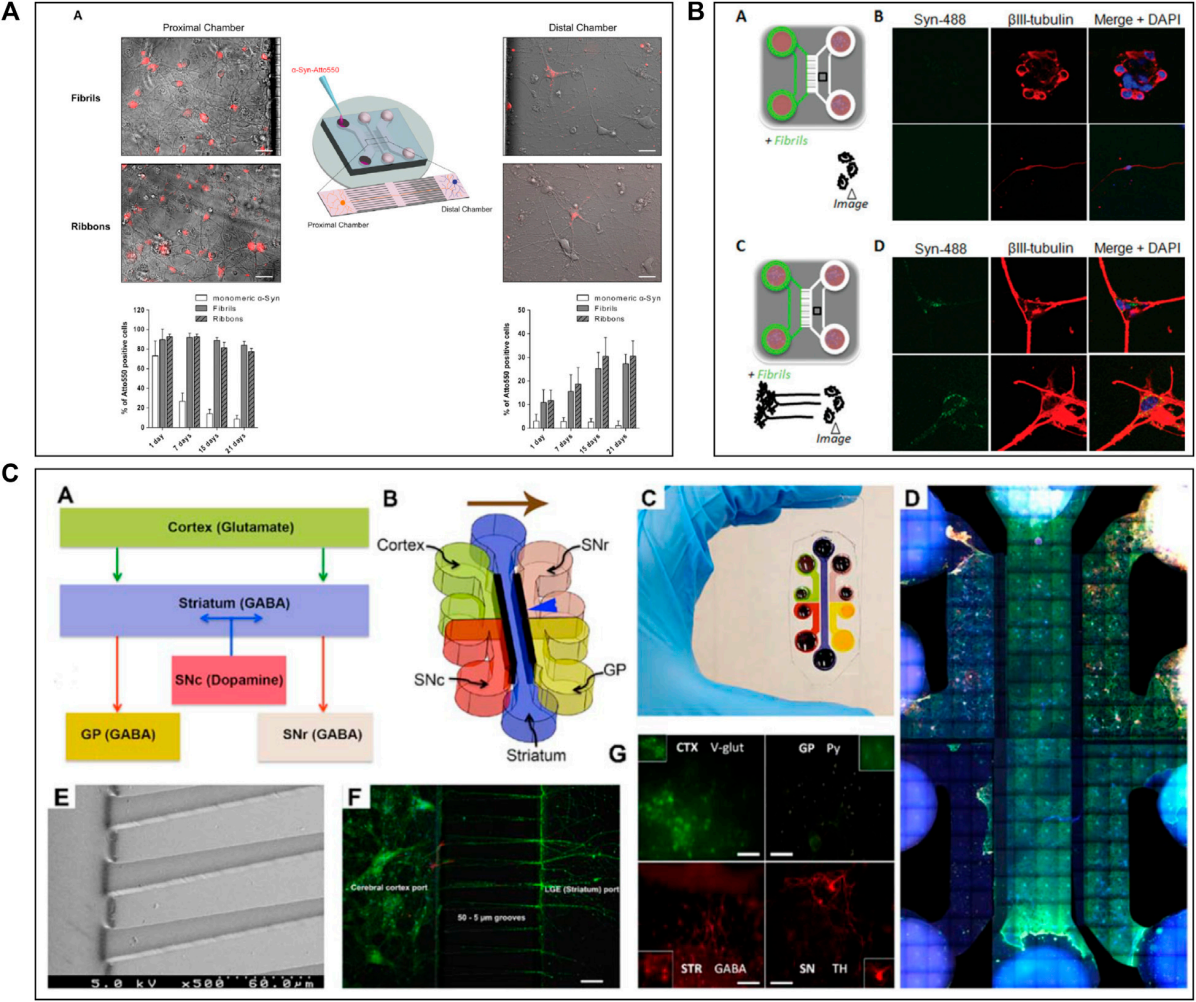
Microfluidic devices used in Amyotrophic Lateral Sclerosis modelling. **(A)** Schematic representation of a 2 compartments microfluidic device for neuro-muscular junction (NMJ) modelling in PDMS on glass coverslips with timeline of experimental procedure in the device. Extracted from Osaki et al. (2018c) (copyright Science advances). **(B)** Co-culture representation of motoneurons and muscular cells in microfluidic device with brightfield and immunofluorescent pictures of co-culture from DIV2 to DIV18, DIV: Days *In Vitro*. Scale bars represent 20 µm Extracted and adapted from Southam et al. (2013) (copyright Journal of Neuroscience Methods). **(C)** Schematic representation of microfluidic device with co-culture; Spinal cord explant and skeletal muscle cells in a device and Superior cervical ganglion and cardiomyocytes in another device with immunofluorescent pictures of cell type and their functional activity. Scale bars represent 20 µm Extracted from Altman et al. (2019) (copyright Journal of Cell Science). **(D)** Representation of a vascular (endothelial cells) and neuronal (motoneurons) networks co-culture in microfluidic device with collagen gel. Extracted from Osaki et al. (2018b) (copyright Scientific Reports).

ALS is also defined as a “prion-like” disease and the degeneration of the NMJ is one of the main hallmarks (Ayers and Cashman, 2018). For the study of ALS mechanisms, scientists have used microfluidic systems allowing the co-culture of motor neurones and myocytes to build accurate *in vitro* NMJ models (Southam et al., 2013; Blizzard et al., 2015; Natarajan et al., 2019; Jongh et al., 2021) (Figure 3). Most researchers have used a two (Park et al., 2013a; Southam et al., 2013; Blizzard et al., 2015; Zahavi et al., 2015; Westergard et al., 2016; Santhanam et al., 2018; Altman et al., 2019) or three (Machado et al., 2019; Spijkers et al., 2021) compartments microfluidic device connected via classical microchannels. The neurites of the motor neurons grew from one compartment up to the compartment that was seeded with myocytes, therefore creating a minimalistic representation of the NMJ. Zahavi et al. (2015) and Blizzard et al. (2015) used a microfluidic device with two compartments and demonstrated the degeneration from myocytes to motor neurons by showing a retrograde transmission of toxic compounds. Other researchers as Santhanam et al. (2018) and Westergard et al. (2016) seeded hiPSC-derived motor neurons and myocytes into the microfluidic device, in order to fabricate a more relevant model using human cells. Santhanam et al. (2018) investigated dose response evaluation of therapeutics to demonstrate pharmacological relevant response while Westergard et al. (2016) have shown toxic protein spreading led to cell death and thus, neurodegeneration.

Neuroinflammation is another hallmark of ALS (Guo et al., 2017; Valko and Ciesla, 2019), with astrocytes and glial cells involvement. Some studies aimed to determine the involvement of glial cells in the development of this disease using microfluidic models (Southam et al., 2013; Machado et al., 2019) (Figure 3B). Machado et al. (2019) used a three-chamber device to study the connection and the activity between motor neurons, astrocytes, and myocytes through optogenetics (Machado et al., 2019). In this study, ALS-mutant co-culture of neurons and astrocytes, both expressing light-sensitive Channelrhodopsin 2 (ChR2), were separately seeded into the two distal chambers, while myofibrils were plated in the central compartment. They also seeded another group of ALS-mutant co-culture that did not express the ChR2. They explored axonal transport and protein aggregation by contrasting ChR2-active and ChR2-inactive neural cells (Machado et al., 2019). Using the same microfluidic device design, Altman et al. (2019) studied moto neurons-muscle cells synapses and mitochondrial motility and localization. Thanks to parallelized microchannels, they were able to actively follow mitochondrial transport within axons and demonstrated that the health of NMJ depends on the accumulation of mitochondria in the motoneurons-muscle cells synapses (Figure 3C).

Recently, some innovative ALS models using microfluidic systems have also been developed by integrating 3D cultures generated with hiPSC-derived cells coming from ALS patients. In 2018, Osaki et al. performed two studies (Osaki et al., 2018c; 2018b) on *in vitro* ALS modelling. In the first study (Osaki et al., 2018c), they seeded hiPSC-derived skeletal myoblasts in one chamber and ChR2-positive hiPSC-derived motor neurons in form of 3D spheroids in the other, connecting both chambers with microchannels to reconstruct a functional NMJ. By activating motor neurons through light, they demonstrated that the muscle contraction initiated by ALS-derived motor neurons was weak, although this phenotype could be pharmacologically reversed (Osaki et al., 2018c) (Figure 3A). In the other study (Osaki et al., 2018b), they investigated the involvement of neurovascular coupling in the progression of motor neuron diseases. They co-cultured 3D motor neuron spheroids and human-derived endothelial cells in a microfluidic device, showing that the presence of vascular cells improved interneuronal connectivity (Osaki et al., 2018b) (Figure 3D).

## Conclusion

In this review, we presented the utility of microfluidic devices and models for the *in vitro* study of NDD pathophysiology and pharmacology. Most of the researchers that have used microfluidic devices used devices with two or three compartments. These devices enabled them to investigate the propagation of peptides or molecules along neurons and synapses, as well as some neuroinflammatory aspects involved in NDD (Chen et al., 2016). These microfluidic architectures could be used for CNS and PNS modelling to investigate molecular mechanisms as well as perform pharmacological studies and drug screening (Nikolakopoulou et al., 2020). More NDD studies with microfluidic systems are progressively using hiPSC to get as close as possible to the human physiology and to reduce the gap between *in vivo* and *in vitro* models (Sosa-Hernández et al., 2018).

Specifically, iPSC technology is used to create cellular models in 3D culture, called organoids, to reproduce human organs including the brain (Lancaster et al., 2013) for *in vitro* brain models (Bassi et al., 2021). The use of human stem cells and iPSCs that have the capacity to give several neural and non-neural cells is essential to create relevant and reproductible models. Organ-on-chip technology, also called microphysiological systems (MPS), allow to mimic multiple organs microenvironment and improve the relevance of *in vitro* model systems (Sung et al., 2019). To exemplify, SNP and SNC cell models can be integrated in the same device to develop complex, yet relevant, neural systems on chip (Anderson et al., 2021). By combining such models, MPS have been developed to co-culture endothelial and neural cells to mimic the BBB (Oddo et al., 2019; Yoon et al., 2021) or with human tissues culture (Holloway et al., 2021). Key features of BBB-on-chip are 2D or 3D co-culture and relevant cellular microenvironment allowing improvements in drug discovery, toxicology, brain research and personalized medicine (Oddo et al., 2019; Yoon et al., 2021).

MPS have also found several applications in different organs modelling, such as gut (Raimondi et al., 2019), liver (Banaeiyan et al., 2017), retina (Achberger et al., 2019), skin (Sutterby et al., 2020) and others reviewed by D.E. Ingber (Ingber, 2022). The microbiota-neurodegeneration hypothesis can be study by the interface between individual organ-on-chip as brain and gut tissues creating a barrier model and study the penetration of neurotoxins through microbiota (Raimondi et al., 2019; Ambrosini et al., 2020; Ceppa et al., 2020). Other applications of MPS includes blood-retinal with epithelial cells and vascular endothelial cells co-culture for the investigation of ophthalmological diseases (Ragelle et al., 2020; Arik et al., 2021).

These multi-organs devices allow the investigation of absorption, distribution, metabolism, elimination and toxicity (ADMET) of drugs and pharmacokinetic-pharmacodynamic (PK-PD) studies and assays (Isoherranen et al., 2019; McAleer et al., 2019; Shinha et al., 2020). Isoherranen et al. (2019) have reviewed the role of organ-on-a-chip technologies in PK-PD studies and presented several multi-organ-on-a-chip including BBB target.

In summary, BoC technologies are increasingly used and standardized in the search for new and relevant *in vitro* models, while aiming at utilizing human cells and thus limiting the use of the animal testing. Major advances are being made in neurodegenerative diseases allowing to improve knowledges of these disorders and providing relevant and reproducible high-throughput drug screening for the emergence of new treatments and hopefully they will allow personalized medicine for every suffering patients (Kankala et al., 2018). Moreover, in the future it can be envisaged that BoC could be coupled with other organ-on-chip, for example lung, liver and BBB, to improve ADMET assays (Herland et al., 2020; Picollet-D’hahan et al., 2021).
