# Supplementary material for: Modeling Neurodegenerative Diseases Using In Vitro Compartmentalized Microfluidic Devices
Source: Front Bioeng Biotechnol. 2022 Jun 24;10:919646. doi: 10.3389/fbioe.2022.919646 (PMC9263267; doi:10.3389/fbioe.2022.919646)
Supplement: Supplementary file 1 [file Table1.DOCX]

Supplementary Material

**Table S1.** Different microfluidic techniques in study of Alzheimer’s disease, Parkinson’s diseases, Lewy’s bodies disease and Amyotrophic Lateral Sclerosis. ACs: Astrocytes; (h)AD: (human) Alzheimer’s disease; ALS: Amyotrophic lateral sclerosis; agg: aggregation; α-syn.: α-synuclein; Bi: Bidirectional; CHO: Chinese Hamster Ovary; Comp.: Compartmentalized; Conn.: Connectivity; DLB: Dementia with Lewy bodies; DPR: dipeptide repeat proteins; EC: endothelial cell; ESC: embryonic stem cell; inf: inflammation; (h)iPSCs: (human) induced pluripotent stem cells; FTD: Frontotemporal dementia; GP: Globus Pallidus; HD: Huntington disease; LBD: Lewy’s bodies disease; (h)MN: (human) motor neuron; N2a: mouse neuroblastoma cells; NMJ: neuromuscular junction; NSC: neural stem cells; mHTT: mutant huntingtin; oligo: oligomer; PD: Parkinson’s disease; Pyk2: Protein tyrosine kinase 2 beta; SN: Substantia Nigra; 3R: three-repeat; Uni: Unidirectional;

| Disease | Injury | Aim of the study | Microfluidic devices | | | Cell type | Drug test. | Rational using devices | Date and ref. |
| --- | --- | --- | --- | --- | --- | --- | --- | --- | --- |
|  |  |  | Type | # nodes | Conn. |  |  |  |  |
| AD | Aβ | Aβ-induced synaptic alteration and neurons death with back propagation on axons in a microfluidic-based reconstructed neuronal network | Comp. | 2 | Uni | Primary cortical and hippocampal neuron of E16 mouse embryos | No | Axonal Transport mechanisms | (Deleglise et al., 2014)  2014 |
|  | Aβ agg. | Dissociation and clearance of metal ion induced Aβ aggregates in microfluidic | Comp. | 2 | Bi | hAβ42 | Yes | Aβ directional transport | (Lee and Park, 2010)  2010 |
|  | Aβ | Role of soluble and bound Aβ on microglial accumulation | Comp. Circular device | 2 | Bi | Human brains + human microglial cells | No | Microglial migration | (Cho et al., 2013)  2013 |
|  | Aβ oligo. or fibrillar | Testing Aβ toxicity | Channel (1 inlet + 4 outlet) | 1 | Bi | Primary hippocampal neurons of E18-21 rat embryos | Yes | Parallelisation drug assay | (Ruiz et al., 2014)  2014 |
|  | Tau | Propagation of phosphorylated tau (high-molecular-weight) | Comp. | 3 | Bi | Primary cortical neuron of E15 mouse embryos | No | Axonal transport and synaptic transmission | (Takeda et al., 2015)  2015 |
|  | Tau | Evaluation of cell-to-cell tau propagation | Comp. | 3 | Bi | Primary hippocampal neurons of E17 rat embryos | No | Synaptic transport mechanisms | (Calafate et al., 2015)  2015 |
|  | Tau (h lentivral) | Neuron-to-neuron transport of Tau protein through a trans-synaptic mechanism | Comp. | 2 | Bi | Rat hippocampal in vivo (brain sections) | No | Axonal transport mechanisms | (Dujardin et al., 2014)  2014 |
|  | Hyper P-Tau  (OA) | Generation of co-pathological states of AD with 2 separated primary cortical cell compartments | Comp. | 3 | Bi | Primary cortical neurons (E19) rat embryos | No | Cellular and molecular propagation mechanisms | (Kunze et al., 2011)  2011 |
|  | Aβ42/p-tau/neuro-inf. | A 3D triculture system modelling neurodegeneration and neuroinflammation in AD | Comp. Circular device | 2 | Bi | hiPSC-NPCs | No | Cellular and molecular propagation mechanisms | (Park et al., 2018)  2018 |
|  | Tg2576 neurons/ Aβ oligomers | Demonstrating a BDNF axonal retrograde signalling in AD model that can be reversed by a pharmacological compound | Comp. | 2 | Bi | Primary cortical neurons E18 rat or E16 mice embryos | Yes | Axonal transport mechanisms and synaptic function Neuronal function | (Poon et al., 2011)  2011 |
|  | FITC-Aβ-1-42 | Transmission of β-amyloid via neuronal connections along axonal membranes | Comp. | 3 | Bi | Primary cortical neurons rat embryos | Yes | Cellular and molecular propagation mechanisms | (Song et al., 2014)  2014 |
|  | 3R and 4R tau | Effect of 2 Tau isoforms (3 repeat and 4 repeat) on mitochondrial axonal transport | Comp. | 2 | Bi | H4 human neuroglioma cells/ Primary cortical neurons E15-17 mouse embyos | No | Axonal transport mechanisms | (Stoothoff et al., 2009)  2009 |
|  | Aβ42 in perfusion | Investigation of localized mechanism in Aβ-induced neurotoxicity by insolating axons from the cell body | Comp. | 3 | Bi | Primary hippocampal neurons from E18 rat | Yes | Aβ localization and subcellular toxicity | (Li et al., 2017)  2017 |
|  | Aβ42 peptide | Characterization of Aβ synaptotoxicity | Comp. | 3 (+1) | Bi | Primary hippocampal and cortical neurons from P0 rats / CHO cell lines with genetic mutation (APP VV717I) | Yes | Aβ toxicity on synapses | (Kilinc et al., 2019)  2020 |
|  | Tau from hAD brains | Detecting and quantification of formation and propagation of Tau aggregates | Comp. | 2 | Bi | Primary cortical neurons from E18 rat | No | Neuronal propagation and axonal transport | (Katsikoudi et al., 2020)  2020 |
|  | Recombinant Tau protein | Studying Tau proteins transport and internalization in neurons | Comp. | 2 | Bi | Primary hippocampal and cortical neurons of E16-20 mouse brain | No | Proteins anterograde and retrograde transport in neurons | (Wu et al., 2013)  2012 |
|  | Recombinant human Tau | Studying tau pathology propagation along neurons | Comp. | 2 | Bi | hiPSC-derived neurons | No | Molecular and cellular mechanisms of Tau transmission | (Usenovic et al., 2015)  2015 |
|  | Human Tau from donor cells | Testing of extracellular tau generated by neurons lead to cell-to-cell spreading | Comp. | 2/3 | Bi | Primary hippocampal and cortical neurons of rat / iPSC- derived cortical neurons | No | Cell-to-cell tau propagation | (Wu et al., 2016)  2016 |
|  | N2a expressing the 4R of Tau | Testing of Tau spreading along neurons via exosomes | Comp. | 2 | Bi | Mouse neuroblastoma cells (N2a)/ Primary cortical neurons from E18 rat embryos | No | Tau neuronal propagation | (Wang et al., 2017)  2017 |
|  | rTg4510 brain extract | Testing of different tau antibodies on the tau spreading along neurons | Comp. | 3 | Bi | Primary cortical neurons from E14-15 mouse embryos | Yes | Tau neuronal propagation | (Nobuhara et al., 2017)  2017 |
| PD | 6OHDA | Generation of an in vitro model of PD with a microfluidic-based neurotoxin concentration gradient | 1 channel | 1 | Uni | PC12 cells | No | Neuronal death based on gradient | (Seidi et al., 2011)  2011 |
|  | α-syn | Immunotherapy against propagation of misfolded α-synuclein and neurodegeneration in PD and PD forms (like dementia with Lewy bodies) | Comp. | 3 | Bi | Primary hippocampal neurons from E16 to E18 mouse brains | Yes | Molecular propagation mechanisms and synaptic integrity | (Tran et al., 2014)  2014 |
|  | α-syn | Propagation of α-synuclein in microfluidic devices corticocortical neuronal networks using hiPSCs | Comp. | 3 | Bi | hiPSC-NPCs | No | Cellular and molecular transport mechanisms and synaptic integrity | (Gribaudo et al., 2019)  2019 |
|  | α-syn | Cell co-culture platform in microfluidic device for the study of the molecular mechanisms (spreading of α-syn and inflammation) of PD and other synucleinopathies | Comp. | 2 | Micro-channel with micro-valves | 1/ Human neuroglioma cells (H4) were co-culture with cells exp α-syn (+GFP)   2/ H4 cells were co-cultured with N9 microglial cells | No | Molecular propagation mechanisms | (Fernandes et al., 2016)  2016 |
|  | α-syn | Characterization and quantification of the axonal transport of α-syn fibrils which could be transferred from axons to neurons with an anterograde transport | Comp. | 2 | Bi | Primary cortical neurons from E17 mice | No | Axonal transport mechanisms | (Freundt et al., 2012)  2012 |
|  | Ø | Visualization of mitochondrial transport in aligned dopaminergic axons with a microdevice platform | Comp. | 2 | Bi | Dopaminergic neurons with GFP from transgenic mice | No | Mitochondrial transport mechanisms | (Lu et al., 2012)  2012 |
|  | α-syn (oligomers) | Induction of axonal dysfunction with α-synuclein oligomers in human iPSC-based models of synucleinopathies | Comp. | 2 | Bi | hiPSC from a PD patient donor/ Human primary astrocyte cell | No | Proteins anterograde and retrograde transport in neurons | (Prots et al., 2018)  2018 |
|  | TTX (blocking Na+) | Evaluation of network connectivity with a pharmacological agent induction blocking neuronal activity in a complex microfluidic device | Comp. | 5 | Uni | Cortical, SN, GP and striatal neurons from E15-16 or E12-13 rat embryos tissues | No | Functional connectivity in a complex network | (Kamudzandu et al., 2019)  2019 |
| PD/AD/HD | α-syn/ Aβ42/ mHTT | Investigation of proteins neuron-to-neuron transmission as one cause of neuronal death | Comp. | 2 | Bi | Primary cortical neurons from E17 mouse embryos | No | Molecular propagation and proteins transport | (Brahic et al., 2016)  2016 |
| DLB | α-syn (Lewy bodies) | Induction of Lewy body pathology leading to synaptic dysfunction and neuron death by exogenous α-synuclein | Comp. | 2 | Bi | Primary hippocampal neurons (E16-18) mouse | No | Molecular propagation mechanisms | (Volpicelli-Daley et al., 2011)  2011 |
| DLB/ PD | α-syn (Lewy bodies from human PD patients) | Using platform combining high content screening (HSC), standard culture and microfluidic tools for characterizing the mechanism of action of Lewy Bodies preparations, and for identifying drugs or biologics capable of combatting α-synuclein toxicity and spreading | Comp. | 2 | Bi | 1/ Cortical neurons from E18 rats  2/ Primary cultures of cerebral cortical astrocytes from P0-02 rats  3/ Lewy bodies from human PD brains | No | Molecular propagation mechanisms (spreading) | (Cavaliere et al., 2017a)  2017 |
| ALS | Disruption of vascular flow or vascular dysfunction | New 3D co-culture with microvascular and neuronal network model in a microfluidic platform to investigate interactions between these 2 systems | Comp. | 29 | Bi | Human iPS-derived endothelial cells / human embryonic stem-derived motor neurons | Yes | Interactions between microvascular and neuronal network model | (Osaki et al., 2018b)  2018 |
|  | H2O2 (oxidative stress) | Neuromuscular co-culture in a compartmentalized microfluidic device to study spatial aspects of GDNF functions | Comp. | 2 | Bi | Primary cultures and spinal cord explants of HB9::GFP mouse / Skeletal myocyte of adult mouse | Yes | Retrograde transport of molecules from muscle to neuron | (Zahavi et al., 2015)  2015 |
|  | ALS-iPSC-derived NSCs with AAV | ALS-on-a-chip model using 3D skeletal muscle from iPSC and optogenetic motor neurons | Comp. | 2 | Bi | hESC-derived NSCs and ALS-iPSC-derived NSCs / humain iPSC-derived skeletal myoblasts / human iECs / mouse myoblasts / hESC-derived NSCs and iPSC derived NSCs from patient with sporadic ALS | Yes | 3D co-culture study (iALS-MNs and iPSC-derived 3D muscle fiber bundles) | (Osaki et al., 2018c)  2018 |
|  | Kainic acid (KA) | Investigation of the specific site of vulnerability of lower motor neurons (with excitotoxin exposure targeted to either cell body or NMJ) | Comp. | 2 | Bi | Primary mouse motor neuron from spinal cord (E13.5)/ C2C12 myoblast cells | No | Co-culture (MN and myocytes) and compartmentalised model of site-specific (somatodendritic and axonal) | (Blizzard et al., 2015)  2015 |
|  | Stem cells from C9orf72-ALS patients | Using different experimental cell culture platforms to find cell-to-ell spreading of DPR via different pathways | Comp. | 2 | Bi | Exosomes from NSC-34 cells / Stem cells from C9orf72-ALS patients | No | Cell-to-cell and proteins (DPR) spreading | (Westergard et al., 2016)  2016 |
|  | hMNs from hiPSCs | Quantifiable functional hNMJ system to establish a platform for generating patient-specific MJ models by including patient-derived iPSCs | Comp. | 2 | Bi | Human spinal cord stem cell derived MNs and human iPSCs derived MN / human myoblasts | Yes | Co-culture (NMJ) | (Santhanam et al., 2018)  2018 |
|  | Ø | Modelling the NMJ with microfluidic primary culture | Comp. | 2 | Bi | Mixed spinal glial cells (P2) / spinal motor neurons (E15) / skeletal myocytes (P2) | Yes) | Co-culture for NMJ model | (Southam et al., 2013)  2013 |
|  | C57BL/6J mice with SOD1G93A mouse strain and TTX treatments | Comparison between NMJ and another synapse function to provide a possible explanation why the NMJ differs from other synapses | Comp. | 2 | Bi | MN-skelatal muscle co-culture and sympathetic neurons-cardiomyocyte | No | Co-culture for NMJ function | (Altman et al., 2019)  2020 |
|  | SOD1G93A mutations on MNs and ACs | In vitro modelling of nerve-muscle connectivity and activity depending on optogenetic entrainment | Comp. | 3 | Bi | MNs and ACs on spheroid with ChR2 active and inactive with myofibrils co-culture | No | Co-culture for NMJ function with active and inactive neuronal populations | (Machado et al., 2019)  2019 |
